# La osteocalcina se asocia con la densidad mineral ósea y los polimorfismos del gen *VDR* en la diabetes tipo 1 y 2

**DOI:** 10.1515/almed-2023-0158

**Published:** 2023-12-13

**Authors:** Carla Ramírez Ruiz, Nerea Varo Cenarruzabeitia, Miriam Martínez Villanueva, Antonio M. Hernández Martínez, José A. Noguera Velasco

**Affiliations:** Departamento de Bioquímica Clínica, Clínica Universidad de Navarra, Madrid, España; Departamento de Bioquímica Clínica, Clínica Universidad de Navarra, Pamplona, España; Departamento de Bioquímica Clínica, Hospital Clínico Universitario Virgen de la Arrixaca, Murcia, España; Departamento de Nutrición y Endocrinología, Hospital Clínico Universitario Virgen de la Arrixaca, Murcia, España

**Keywords:** hueso, marcadores de remodelado óseo, diabetes mellitus, osteocalcina, osteoporosis, polimorfismos de *VDR*

## Abstract

**Objetivos:**

El metabolismo óseo se encuentra alterado en la diabetes mellitus (DM). El objetivo de este estudio es evaluar la relación entre los marcadores de remodelado óseo (MRO), los polimorfismos en el gen receptor de la vitamina D (*VDR*) y la densidad mineral ósea (DMO) en la DM tipo 1 (T1D) y tipo 2 (T2D).

**Métodos:**

Se incluyó a 165 pacientes (53 T1D y 112 T2D). La DMO se midió mediante absorciometría de rayos X de energía dual (DEXA). Se realizó un análisis de la osteocalcina (OC) en plasma, *beta-CrossLaps* (β-CTX), propéptido aminoterminal del procolágeno tipo 1 (P1NP) y los polimorfismos en el gen *VDR*.

**Resultados:**

Se incluyó a 53 pacientes con T1D (41 años (31–48)) y 112 con T2D (60 años [51–66]). No se observaron diferencias estadísticamente significativas en relación a la DMO. Los pacientes con T1D presentaron niveles superiores de OC (p<0,001) y P1NP (p<0,001). Las áreas bajo la curva para la predicción de patología ósea para la OC fueron 0,732 (p=0,038) en T1D y 0,697 (p=0,007) en T2D. Se observó una relación estadísticamente significativa entre el alelo A de *BsmI* (p=0,03), el alelo A de *ApaI* (p=0,04) y el alelo C de *Taql* (p=0,046) y una menor DMO. Así mismo, se encontró una correlación significativa entre los niveles elevados de OC y el alelo G de *BsmI* (p=0,044), el alelo C de *ApaI* (p=0,011), el alelo T de *Taql* (p=0,006) y el alelo C de *FokI* (p=0,004).

**Conclusiones:**

El elevado valor predictivo negativo del punto de corte de la OC indica que la OC podría ser útil a la hora de descartar el riesgo de pérdida ósea, lo que permitiría diseñar un tratamiento personalizado para prevenir dicha patología.

## Introducción

La diabetes mellitus (DM) se caracteriza por niveles elevados de glucosa en sangre que, con el tiempo, no sólo afectan gravemente al corazón, los vasos sanguíneos y otros órganos, sino que está asociada a un mayor riesgo de fracturas. Diversos mecanismos como la hiperglucemia, la insulina, el estrés oxidativo, y el déficit de vitamina D, entre otros, que ocurren en ambos tipos de diabetes, pueden afectar a la fuerza y el metabolismo óseo [1]. Por lo tanto, es necesario realizar pruebas de cribado de osteoporosis en pacientes con diabetes.

Según la Organización Mundial de la Salud (OMS), el método de referencia para diagnosticar osteoporosis u osteopenia, es la evaluación de densidad mineral ósea (DMO) [2]. Sin embargo, dicha prueba presenta algunas limitaciones, ya que no proporciona información sobre la calidad ósea. Además, el diagnóstico de osteoporosis en pacientes diabéticos supone un reto, ya que, paradójicamente, los DM tipo 2 (T2D) tienden a tener una DMO normal o elevada, pero con un mayor riesgo de fracturas [3].

Por esta razón, se requieren otros enfoques diagnósticos para identificar el nexo entre la DM y la osteoporosis. Los marcadores de remodelado óseo (MRO) han surgido como alternativa a la evaluación de la DMO, ya que reflejan la actividad metabólica del hueso. Aunque no están validados para el diagnóstico, los niveles elevados de MRO predicen la pérdida ósea y son útiles para evaluar la respuesta al tratamiento.

La mayoría de los estudios de MRO se han realizado en mujeres posmenopáusicas o en hombres de edad avanzada. Los estudios que evalúan los marcadores óseos de síntesis y resorción en DM tipo 1 (T1D) y T2D han obtenido resultados contradictorios, ya que mientras algunos de estos estudios describen niveles reducidos de MRO, otros no muestran diferencias significativas [4], [5], [6]. Parece que los pacientes diabéticos muestran un patrón disociativo, puesto que los cambios en la formación ósea no se ven reflejados en cambios en la resorción [7]. Es posible encontrar niveles bajos de MRO con una DMO baja, normal o incluso elevada en pacientes con mayor riesgo de fractura, lo que sugiere que los intervalos de referencia tradicionales pueden no ser adecuados para la toma de decisiones clínicas [8]. Por lo tanto, se necesitan más estudios para establecer la utilidad de los MRO como herramienta de cribado en la población diabética.

Por otro lado, los estudios familiares y de gemelos sugieren que la DMO tiene un alto grado de heredabilidad [9]. Se han realizado multitud de estudios para identificar los genes que contribuyen al desarrollo y mantenimiento de la masa ósea. El gen del receptor de la vitamina D *(VDR)* ha sido objeto de numerosos estudios, debido al papel esencial que desempeña la vitamina D en el metabolismo óseo [10, 11]. Los factores genéticos también son de gran interés en los pacientes diabéticos, pero en la actualidad los estudios son insuficientes, varían entre poblaciones y son limitados los que combinan polimorfismos del gen *VDR* y el análisis de los MRO [12, 13]. Se necesitan más conocimientos sobre la contribución genética al metabolismo óseo en la diabetes y sobre los factores que pueden identificar a los pacientes con alto riesgo de pérdida ósea y, por tanto, con mayor probabilidad de requerir tratamientos en el futuro.

En este contexto, los objetivos del presente estudio fueron: (1) estudiar la asociación entre los niveles de MRO (osteocalcina (OC)), *beta-CrossLaps* (β-CTX) y propéptido aminoterminal del procolágeno tipo 1 (P1NP) con la DMO en pacientes con T1D y T2D; (2) proporcionar información sobre si las variantes genéticas de los polimorfismos del gen *VDR* predisponen a los pacientes con diabetes a desarrollar osteoporosis.

## Materiales y métodos

### Sujetos y estudios clínicos

Llevamos a cabo un estudio prospectivo en el Hospital Clínico Universitario Virgen de la Arrixaca (Murcia, España). La población de estudio estaba compuesta por 165 pacientes caucásicos diagnosticados de DM (53 con tipo 1 y 112 con tipo 2) (edad, 18–70 años) reclutados en el Departamento de Endocrinología.

Las personas con enfermedades oncológicas, diabetes secundaria a otras patologías, enfermedades o condiciones que afectan el recambio óseo fueron excluidas del estudio. También se excluyeron pacientes que estuvieran tomando glucocorticoides orales, terapia de reemplazo hormonal o cualquier tratamiento que pudiera interferir con el metabolismo óseo.

En el momento del reclutamiento, se recopilaron datos relativos a complicaciones derivadas de la diabetes, antecedentes familiares de fracturas, hábito tabáquico, consumo de alcohol, actividad física, exposición al sol, consumo de café, tiempo de evolución de la diabetes y tratamiento. Así mismo, se midió la altura y el peso corporal y se calculó el índice de masa corporal (IMC). Se recopilaron datos sobre complicaciones microvasculares (nefropatía, retinopatía y neuropatía) y complicaciones macrovasculares (infarto de miocardio, accidente cerebrovascular y enfermedad vascular periférica).

Todos los sujetos reclutados dieron su consentimiento informado por escrito. El estudio se realizó de acuerdo con la Declaración de Helsinki, y el protocolo fue aprobado por el Comité de Ética local.

### Densitometría

La medición de DMO se realizó mediante absorciometría de rayos X de energía dual (DEXA) en la columna lumbar (L1–L4) y en los tres puntos de la cadera derecha (cuello femoral) con el dispositivo Lunar DEXA, DPX-L. La osteopenia se definió según los criterios de la OMS, con una puntuación T (*T-score*) para columna lumbar o cuello femoral entre – 1 DE y – 2,5 DE. La osteoporosis se definió con un *T-score* en columna lumbar o cuello femoral inferior o igual a – 2,5 DE para las mujeres posmenopáusicas y los hombres mayores de 50 años. Para las mujeres premenopáusicas y los hombres menores de 50 años, la osteoporosis se diagnosticó con una puntuación Z (*Z-score*) de la DMO igual o inferior a – 2 DE en la columna lumbar o el cuello femoral [14].

### Análisis bioquímicos

Para evitar la variación diurna, las muestras de sangre se recogieron tras ayuno nocturno entre las 8:00 y las 10:00 en tubos vacutainer, conservando una alícuota a −80 °C para su posterior análisis. La medición de OC, β-CTX y P1NP se realizó en el laboratorio de bioquímica del Hospital Clínico Universitario Virgen de la Arrixaca mediante quimioluminiscencia (ECLIA) en un analizador Cobas E411 (Roche Diagnostics). Los coeficientes de variación intra e interensayo fueron inferiores al 5 %. Los límites de detección fueron 0,50 μg/L para la OC, 0,01 μg/L para el β-CTX y 5 μg/L para el P1NP.

### Polimorfismos

Se seleccionaron cuatro polimorfismos de un solo nucleótido (SNP) rs1544410, rs7975232, rs731236 y rs2228570, que son algunos de los polimorfismos más estudiados del gen *VDR*, en concreto, *BsmI, ApaI, TaqI* y *FokI* respectivamente. Las frecuencias genotípicas y alélicas de la población de estudio se compararon con las de la población de referencia (población europea del Proyecto 1,000 Genomes y su subpoblación IBS (población ibérica española)), extraídas de ensembl.org.

El ADN genómico se aisló y purificó utilizando el kit de extracción CLART^®^MetaBone (GENOMICA) y se conservó a −20 °C hasta su posterior análisis. El genotipado se realizó mediante la reacción en cadena de la polimerasa a tiempo real con sondas HybProbe utilizando el analizador LightCycler2.0^®^ (Roche Diagnostics^®^).

### Análisis estadísticos

El análisis estadístico se realizó con el programa IBM SPSS Statistics 23.0 (IBM, New York, NY, EE.UU). La distribución normal de las muestras se comprobó mediante la prueba de Shapiro-Wilk. Las diferencias entre grupos se evaluaron mediante la prueba t de Student para las variables con distribución normal, y con la prueba U de Mann Withney para aquellas con distribución no normal. Para las variables categóricas se empleó la prueba de Chi cuadrado. Las correlaciones observadas en el análisis univariante se analizaron mediante las pruebas de correlación de Pearson y Spearman. Para analizar las relaciones independientes de los MRO, se realizó el análisis de regresión lineal multivariante. Se realizó un análisis de curva ROC para determinar el rendimiento diagnóstico de los MRO. Se utilizó el índice de Youden para determinar un punto de corte óptimo para la detección de patología ósea a través de los MRO.

Los datos se resumieron como media ± desviación estándar (DE) para las variables cuantitativas y, como frecuencia, para las variables cualitativas. Se comprobó el equilibrio de Hardy–Weinberg en la distribución de genotipos mediante la prueba de chi-cuadrado. El efecto de los genotipos sobre la DMO se evaluó mediante el análisis de varianza para medidas repetidas. La significación estadística se estableció en p<0,05.

## Resultados

### Características demográficas y clínicas basales

En la Tabla 1 se describen las características demográficas y clínicas basales del grupo de estudio. Como era de esperar, se observaron diferencias estadísticamente significativas entre los dos tipos de DM, en la edad, los años de evolución de la enfermedad, la presencia de obesidad (p<0,001), de enfermedad isquémica cardíaca (p<0,01), hipertensión (p<0,001), dislipidemia (p<0,001) y tratamiento (p<0,001). En comparación con los pacientes con T1D, aquellos con T2D presentaban un IMC (p<0,001) y una edad (p<0,001) significativamente mayor, y una evolución más corta de la diabetes (p<0,001).

**Tabla 1:** Características demográficas y clínicas de la población de estudio.

Variable	T1D (n=53)	T2D (n=112)	Valor p
Edad, años	41 (31–48)	60 (51–66)	<0,001
Duración, años	16 (12–18)	12 (7–18)	<0,001
Obesidad, %	15,1	75,9	<0,001
Tipo de obesidad, %			
Bajo peso	3,8	0	p<0,001
Peso normal	47,2	6,3	
Sobrepeso	34	17,9	
Obesidad tipo 1	13,2	41,1	
Obesidad tipo 2	0	24,1	
Obesidad tipo 3	1,9	10,7	
Comorbilidades, %			
Microangiopatía	5,7	5,4	ns
Nefropatía	26,4	24,1	ns
Retinopatía	30,2	26,8	ns
Accidente cerebrovascular	0	0,9	ns
Neuropatía	18,9	14,3	ns
Enfermedad cardíaca isquémica	0	11,6	0,01
Enfermedad arterial periférica	3,8	3,6	ns
Hipertensión	9,4	59,8	<0,001
Dislipidemia	18,9	75	<0,001
Fumadores, %	30,2	25	ns
Exposición solar, %			
Muy baja	2,3	8,6	ns
Suficiente	25	31,2	ns
Elevada	72,7	60,2	ns
Alcohol, %	0	0,9	ns
Actividad física, %			
Sedentarismo	15,9	30,2	ns
Moderada	59,1	48,1	
Moderadamente activa	18,2	17,9	
Activa	6,8	3,8	
Historia de fracturas osteoporóticas, %	20,8	30,4	ns
Antecedentes personales de fracturas osteoporóticas, %	13,2	12,5	ns
Artritis reumatoide, %	1,9	3,6	ns
Medicación, %			
Metformina	5,7	77,7	<0,001
Inhibidores de DPP4	0,0	45,5	<0,001
Secretagogos	0,0	22,3	<0,001
Glitazonas	0,0	11,6	<0,001
Agonistas GLP1	1,9	26,8	<0,001
Insulina	94,3	64,3	<0,001

Los datos se presentan como medianas (rango intercuartílico [ICR]) para las variables no paramétricas. p para la diferencia entre los pacientes con diabetes tipo 1 y tipo 2. T1D, diabetes tipo 1; T2D, diabetes tipo 2; ns, no significativa.

No se encontraron diferencias entre ambos grupos en relación al sexo, artritis reumatoide, hábito tabáquico, consumo de alcohol, exposición solar, actividad física, antecedentes personales o familiares de fracturas, así como otras comorbilidades (microangiopatía, nefropatía, retinopatía, accidente cerebrovascular, neuropatía y enfermedad arterial periférica).

### Densidad mineral ósea

No se encontraron diferencias estadísticamente significativas entre los dos grupos, en relación a la DMO, evaluada mediante *T-score* y *Z-score* femoral y lumbar (Tabla 2).

**Tabla 2:** Densidad mineral ósea y marcadores de remodelado óseo en los dos grupos de pacientes diabéticos.

Variable	T1D (n=53)	T2D (n=112)	Valor p
T-score cadera, DE	0,14 ± 1,0	0,14 ± 1,0	ns
T-score lumbar, DE	0,28 ± 1,62	0,98 ± 1,74	ns
Z-score cadera, DE	0,71 ± 1,08	0,8 ± 1,43	ns

Masa ósea normal, % (n)	79,2 (42)	73,1 (82)	ns
Baja masa ósea, % (n)	20,8 (11)	22,6 (25)	ns
Osteoporosis, % (n)	0 (0)	4,3 (5)	ns

HbA_1c_, %	7,92 ± 1,24	7,38 ± 1,27	0,022
Glucosa, mg/dL	160,34 ± 81,9	144,99 ± 39,28	ns
PTH, pg/mL	25,52 ± 8,4	31,17 ± 18,15	ns
Vitamina D, ng/mL	21,71 ± 7,09	19,09 ± 6,74	0,029
Creatinina, mg/dL	0,81 ± 0,17	0,93 ± 0,41	0,016
Colesterol, mg/dL	190,43 ± 35,25	172,47 ± 32,08	0,001
Triglicéridos, mg/dL	85,25 ± 44,27	152,12 ± 79,05	<0,001
OC, µg/L	17,65 ± 7,29	12,73 ± 6,23	<0,001
β-CTX, µg/L	0,31 ± 0,19	0,26 ± 0,18	ns
P1NP, µg/L	55,17 ± 37,25	37,52 ± 17,72	<0,001

Los datos se presentan como medias ± DE para las variables continuas y como medianas (rango intercuartílico [IRC]) para las variables no paramétricas. p para la diferencia entre los pacientes con diabetes tipo 1 y tipo 2. T1D, diabetes tipo 1; T2D, diabetes tipo 2; DE, desviación estándar; ns, no significativa.

A continuación, se clasificó a los pacientes según los criterios de la OMS para el diagnóstico de la osteoporosis. Así, 42 pacientes (79,2 %) con T1D presentaron una DMO normal, 11 pacientes (20,8 %) tenían una masa ósea baja y ninguno de ellos tenía osteoporosis. Entre los pacientes con T2D, 82 (73,1 %) tenían una DMO normal, 25 pacientes (22,6 %) presentaban una masa ósea baja y 5 (4,3 %) eran osteoporóticos. No se observaron diferencias estadísticamente significativas entre los dos grupos, en cuanto a la presencia de osteoporosis u osteopenia.

Dado que muy pocos pacientes en los dos grupos padecían osteoporosis, agrupamos a los que presentaron osteopenia y/o osteoporosis en un único grupo (denominado ‘patología ósea’) y se compararon todos los parámetros clínicos y analíticos con los de pacientes sin patología ósea.

### Marcadores de remodelado óseo

**Figura 1:**
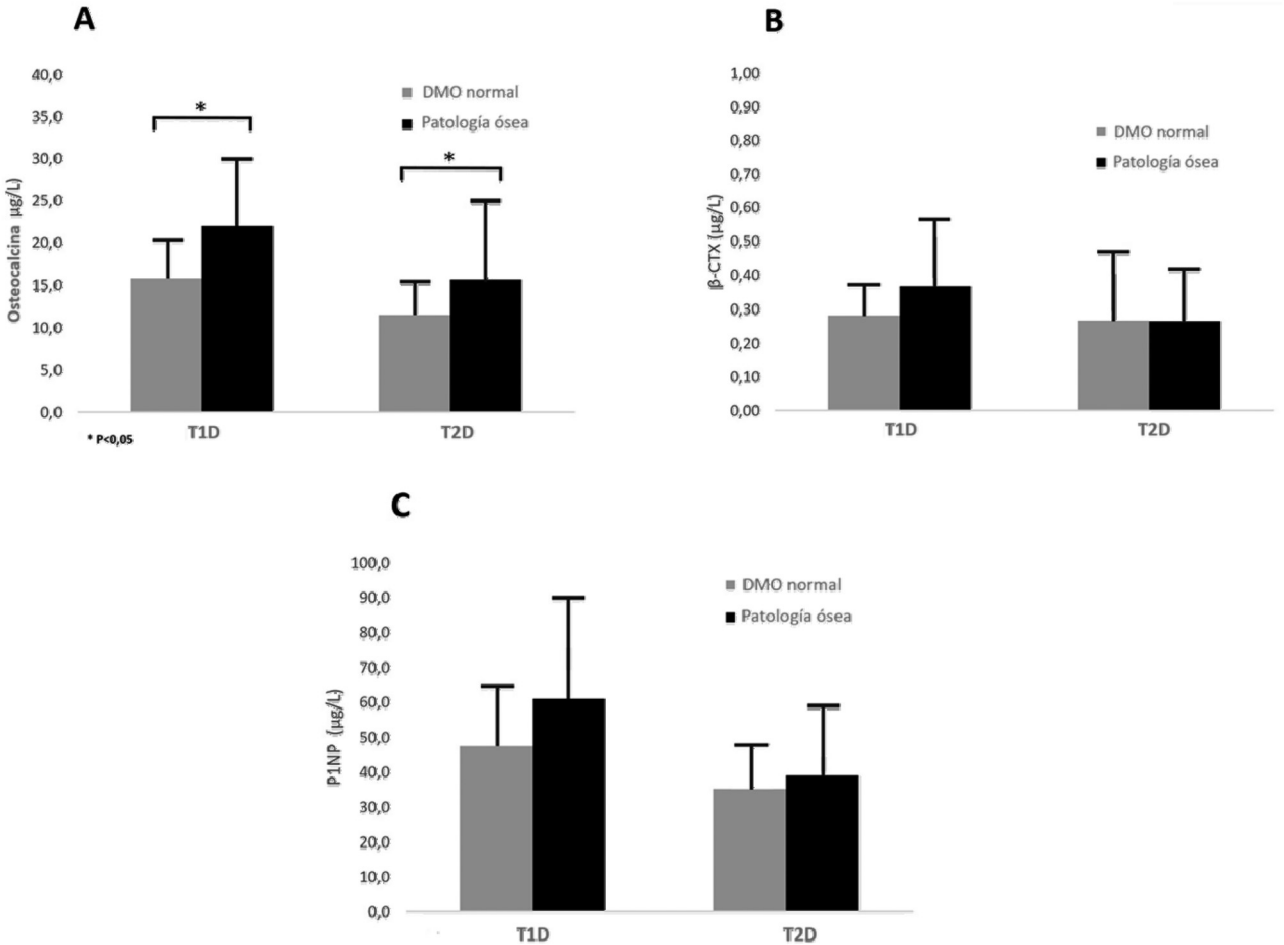
Niveles de los marcadores de remodelado óseo OC (A), β-CTX (B) yP1NP (C) en la T1D y TD2 en pacientes con densidad mineral ósea normal, frente a los pacientes con patología ósea (osteopenia u osteoporosis) en el momento del reclutamiento. Las barras representan la media ± SEM. * Los valores p indican diferencias significativas (p < 0.05).

Se compararon los MRO en pacientes con patología ósea con aquellos con una DMO normal. Tanto en la T1D como en la T2D, se detectaron concentraciones de OC significativamente superiores en los pacientes que presentaban patología ósea en comparación con los que tenían una DMO adecuada (T1D: 15,8 ± 5,1 vs. 22,0 ± 8,4 μg/L, p=0,03 y T2D: 11,5 ± 4,3 vs. 15,7 ± 9,6 μg/L, p=0,021). P1NP en T1D (47,5 ± 18,4 vs. 61,0 ± 30,3 μg/L) y T2D (35,1 ± 13,2 vs. 39,0 ± 20,4 μg/L) y β-CTX en T1D (0,28 ± 0,1 vs. 0,37 ± 0,2 μg/L) y T2D (0,27 ± 0,21 vs. 0,26 ± 0,16 μg/L) tendieron a ser mayores en pacientes con patología ósea, aunque este cambio no alcanzó significación estadística (Figura 1).

Cabe señalar que se encontraron niveles significativamente más altos de los marcadores de formación ósea OC (p<0,001) y P1NP (p<0,001) en pacientes con T1D en comparación con T2D (Tabla 2). Por otro lado, no se encontraron diferencias estadísticamente significativas en los niveles de β-CTX entre ambos grupos. Un número significativo de pacientes tuvieron niveles de OC por debajo de los valores de referencia poblacionales en el momento del reclutamiento, 28 (53 %) T1D y 91 (81 %) T2D.

Se analizaron las posibles asociaciones entre los MRO y otros marcadores analíticos y clínicos. Cabe destacar que, en los pacientes con T1D, se observó una correlación inversa y significativa entre los niveles de OC y la DMO (*Z-score* cadera: r=−0,509; p=0,016, *T-score* cadera: r=−0,399; p=0,021 y *T-score* lumbar: r=−0,329; p=0,022). Asimismo, en pacientes con T2D, los niveles de OC y β-CTX se correlacionaron negativamente con el *T-score* cadera (r=−0,327; p=0,006 y r=−0,238; p=0,048 respectivamente).

Así mismo, observamos una correlación significativa entre los niveles de OC y el metabolismo de la glucosa. En pacientes con T1D, la OC mostró una correlación significativa e inversa con los niveles de hemoglobina glicosilada (HbA_1c_%: r=−0,343; p=0,020). En la T2D, todos los MRO se asociaron negativamente con la glucemia en ayunas (OC: r=−0,229; p=0,018, β-CTX: r=−0,283; p=0,003 y P1NP: r=−0,197; p=0,042) y β-CTX con HbA_1c_ (HbA_1c_%: r=−0,374; p<0,001).

A continuación, evaluamos el rendimiento analítico de cada MRO, así como los puntos de corte con el mejor poder discriminatorio para detectar patología ósea (Tabla 3). Las áreas bajo la curva (AUC) de β-CTX y P1NP para la detección de patología ósea no fueron significativas ni en T1D ni en T2D. El análisis multivariante mostró que la OC era un factor predictivo independiente de patología ósea (*odds ratio* [OR]: 1,16; CI:1,05–1,28; p=0,003).

**Tabla 3:** Puntos de corte y AUC de osteocalcina para detectar patología ósea,

	Punto corte,µg/L	Sensibilidad, %	Especifidad, %	VPP, %	VPN, %	AUC	IC 95 %	Valor p
T1D	20,54	67	86	67	86	0,732	0,52–0,94	0,038
T2D	12,88	65	79	57	84	0,697	0,57–0,80	0,007

T1D, diabetes tipo 1; T2D, diabetes tipo 2; VPP, valor predictivo positivo; VPN, valor predictivo negativo; AUC, área bajo la curva; IC, intervalo de confianza.

### Polimorfismos en el gen receptor de la vitamina D

#### Frecuencias alélicas y genotípicas de *VDR*

En la Tabla 4 se muestran las frecuencias genotípicas y alélicas de los polimorfismos en el gen *VDR* en toda la población y los subgrupos de población (T1D y T2D), comparadas con la población de control (población europea del Proyecto 1,000 Genomes y su subpoblación ibérica española (IBS)). La distribución de los genotipos coincidió con lo esperado, según el equilibro de Hardy-Weinberg. Se registraron las mayores frecuencias de genotipos heterocigotos para todos los polimorfismos examinados.

**Tabla 4:** Comparación de las frecuencias genotípicas y alélicas entre los diferentes grupos de estudio y la población control.

SNP	Población control		Todos los pacientes			T1D			T2D		
	EUR	IBS
	FG	FG	FG	p^a^	p^b^	FG	p^a^	p^b^	FG	p^a^	p^b^
***BsmI* (rs1544410)**											
Genotipo, G/A											
G/G	37	31	32,2	ns	ns	33,3	ns	ns	31,4	ns	ns
A/G	45	50	48,3			50			47,1		
A/A	18	19	19,5			16,7			21,6		
Alelo											
G	60	56	53,5	ns	ns	54,3	ns	ns	53	ns	ns
A	40	44	46,5			45,7			47		
***ApaI* (rs7975232)**											
Genotipo, C/A											
C/C	23	23	22	<0,001	<0,001	21,1	<0,001	<0,05	22,6	<0,001	<0,001
A/C	34	36	51,6			57,9			47,2		
A/A	43	39	26,4			21,1			30,2		
Alelo											
C	45	43	48,6	ns	ns	50	ns	ns	47,5	ns	ns
A	55	57	51,4			50			52,6		
***TaqI* (rs731236)**											
Genotipo, T/C											
T/T	38	33	34,4	ns	ns	35,9	ns	ns	33,3	ns	ns
T/C	44	48	48,9			51,3			47,1		
C/C	18	19	16,7			12,8			19,6		
Alelo											
T	60	57	54,9	ns	ns	58,3	ns	ns	54,9	ns	ns
C	40	42	45,1			41,6			45,1		
***FokI* (rs2228570)**											
Genotipo, C/T											
T/T	16	13	7,8	ns	ns	10	ns	ns	6	<0,05	ns
C/T	44	39	41,1			42,5			40		
C/C	40	48	51,1			47,5			54		
Alelo											
T	38	33	34,7	ns	ns	36,8	ns	ns	36,8	ns	ns
C	62	67	65,4			63,2			63,2		

Los datos se presentan como porcentajes. ^a^p: para las diferencias entre los datos de los pacientes control (población europea del proyecto 1,000 Genomas) y ^b^p: para las diferencias entre los datos de los pacientes control (subpoblación europea (población ibérica de España)) y los pacientes con T1D y T2D. EUR, Europea; IBS, Ibérica, FG, frecuencia genotípica; T1D, diabetes tipo 1; T2D, diabetes tipo 2; ns, no significativa.

No se encontraron diferencias estadísticamente significativas entre los pacientes con T1D y T2D en las frecuencias genotípicas y alélicas de los SNP estudiados (excepto para *ApaI*). Así, a continuación, se evaluaron los polimorfismos agrupando la T1D y la T2D en un único grupo. Observamos una relación estadísticamente significativa entre el polimorfismo *ApaI* y la DM. El genotipo A/C fue más frecuente en pacientes con T1D (p<0,001) y en pacientes con T2D (p<0,001), en comparación con la población control. Hallamos una asociación significativa entre el polimorfismo *FokI* y la T2D cuando se comparó con la población europea, pero no cuando se comparó con la subpoblación ibérica (Tabla 4).

#### Asociación entre el polimorfismo *VDR*, la densidad mineral ósea y los marcadores de remodelado óseo

##### BsmI

**Figura 2:**
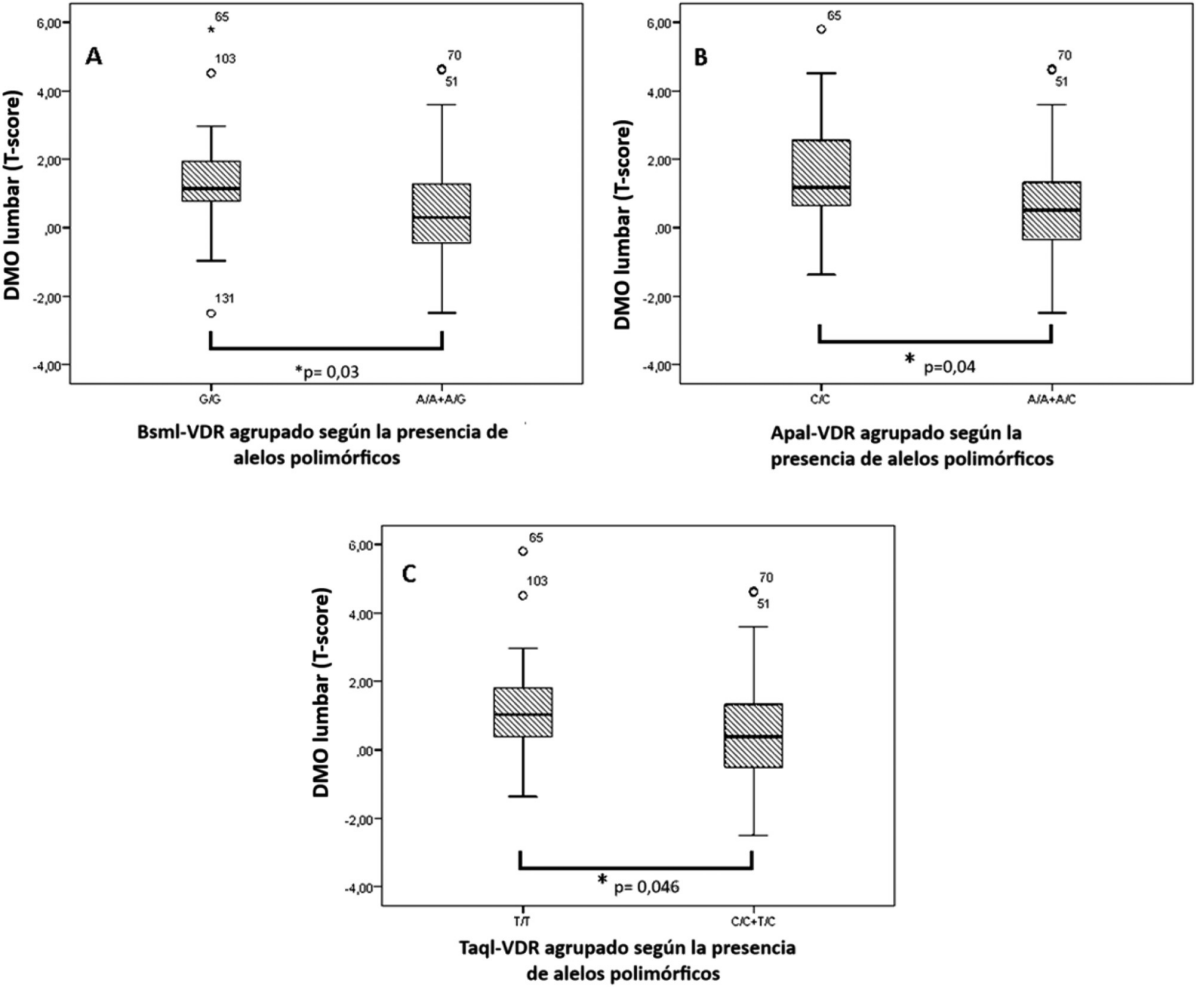
Niveles de DMO (T-score lumbar) según la presencia de los SNP *BsmI* (A), *ApaI* (B), y *TaqI* (C) en el gen *VDR* agrupados de acuerdo con la presencia de alelos polimórficos. Los datos se presentan como medias ± DE. * p indica las diferencias significativas (p < 0,05).

La DMO lumbar (*T-score*) fue significativamente menor en los pacientes con el alelo A (modelo recesivo (GG vs. A/A+A/G) (1,36 ± 1,16 vs. 0,50 ± 1,54; p=0,03)) (Figura 2). Del mismo modo, observamos una correlación entre los MRO y el polimorfismo *BsmI*. Las concentraciones de OC y β-CTX fueron significativamente superiores en los portadores del alelo G frente a los no portadores (modelo dominante (G/G+G/A vs. A/A)), para la OC (15,45 ± 7,98 vs. 12 ± 6,68 μg/L; p=0,044) y para β-CTX (0,31 ± 0,23 vs. 0,19 ± 0,09 μg/L; p=0,016). Del mismo modo, se confirmó una correlación entre el genotipo *BsmI* y los niveles de β-CTX (G/G=0,28 ± 0,17 μg/L; A/G=0,34 ± 0,26 μg/L; A/A=0,19 ± 0,09 μg/L (p=0,027)).

##### ApaI

Se observó una menor DMO lumbar (*T-score*) en presencia del alelo A (modelo recesivo (CC vs. A/A + A/C) (1,53 ± 1,98 vs. 0,56 ± 1,44; p=0,04) (Figura 2). Este SNP también se asoció con los niveles de OC. Se observó una diferencia estadísticamente significativa en los niveles de OC entre los diferentes genotipos (C/C=13,0 ± 6,04 μg/L; A/C=14,05 ± 5,02 μg/L; A/A=9,90 ± 3,21 μg/L (p=0,031)), siendo dichas concentraciones significativamente superiores en los portadores del alelo C (modelo dominante (C/C+C/A vs. A/A) (13,73 ± 5,29 vs. 9,89 ± 3,21 μg/L; p=0,011)).

##### TaqI

Los portadores del alelo C de *Taql* (modelo recesivo (T/T vs. T/C + C/C) presentaron una DMO (*T-score*) significativamente menor (1,27 + 1,54 vs. 0,49 ± 1,62; p=0,046) (Figura 2). Asimismo, este SNP se asoció con los niveles de OC (T/T=14,05 ± 5,82 μg/L; T/C=12,86 ± 4,34 μg/L; C/C=8,97 ± 2,87 μg/L (p=0,021)). Los individuos con alelo T (modelo dominante T/T+T/C vs. C/C) tenían niveles de OC significativamente más altos que los que no portadores (13,40 ± 5,05 vs. 8,97 ± 2,87 μg/L; p=0,006).

##### FokI

No se observó ninguna relación entre *FokI* y la DMO, sin embargo, los niveles de OC fueron significativamente más altos en los pacientes con el alelo C (modelo recesivo T/T frente a C/C + C/T (11.13±0.89 vs. 15.18 ± 8.17 μg/L; p=0.004)).

La relación entre los polimorfismos del gen *VDR* con la DMO y los MRO se proporcionan en el material suplementario (Tabla Suplementaria 1).

## Discusión

Los principales hallazgos del presente estudio son los siguientes: (1) No se observaron diferencias en la DMO entre pacientes con T1D o T2D, pero los marcadores de formación OC y P1NP fueron más bajos en T2D frente a T1D; (2) un elevado porcentaje de pacientes diabéticos presentó niveles de OC por debajo de los valores de referencia; (3) valores bajos de OC se asociaron a una mayor DMO y a un peor control glucémico; (4) los alelos A de *BsmI*, A de *ApaI* y C de *TaqI* se asociaron a una menor DMO lumbar y (5) los alelos G de *BsmI*, C de *ApaI* y T de *TaqI* y C de *FokI* se asociaron a niveles más altos de OC.

### Densidad mineral ósea en pacientes con diabetes tipo 1 y tipo 2

En el presente estudio no encontramos diferencias en la DMO, ni en la prevalencia de patología ósea entre pacientes con T1D y T2D, a pesar de la diferencia de edad, un factor que influye en la masa ósea del individuo. Estos resultados concuerdan con el estudio de Leidig-Bruckner y col. [15], donde la prevalencia de osteoporosis en ambos sexos fue equivalente en T1D y T2D, pero menor en T2D en comparación con la población sana. El estudio de Díaz-Curiel y col. en una cohorte de mujeres sanas españolas reveló una prevalencia de la osteopenia (13 %) ligeramente inferior a la hallada en nuestro estudio en pacientes de la misma edad con T1D [16]. Esto podría deberse a que la T1D se desarrolla años antes de alcanzar el pico de masa ósea, y, por tanto, el cambio en el metabolismo debido a la presencia de la enfermedad, afecta al desarrollo óseo. En la T2D, se observó una prevalencia de la osteopenia inferior a la esperada para el mismo rango de edad en la población sana (22,6 % frente a 42–50 %), lo que sugiere que el uso de DEXA puede no ser la mejor herramienta a la hora de clasificar a los pacientes diabéticos [16].

### Utilidad de los marcadores de remodelado óseo en pacientes diabéticos

Diferentes estudios han evaluado la capacidad de la MRO para predecir la tasa de pérdida ósea, observando que niveles más elevados de MRO se asocian a una mayor tasa de pérdida ósea. Nuestros resultados concuerdan con trabajos previos que describen, paradójicamente, que los niveles de OC son inferiores al rango de referencia tanto en la T1D como en la T2D, lo que refleja una menor formación ósea en ambos tipos [9], [10], [11], [12].

Nuestro estudio muestra que los niveles de OC y P1NP eran más bajos en los pacientes con T2D que en los pacientes con T1D, sin diferencias en los niveles de β-CTX entre ambos grupos. Asimismo, estos resultados concuerdan con trabajos previos [17], [18], [19], [20], [21]. La insulina tiene un efecto anabólico sobre el hueso, por lo que el hallazgo de niveles inferiores de OC en la T2D podría estar relacionado con la resistencia a la insulina que caracteriza a este tipo de pacientes.

Este estudio amplía estos hallazgos al demostrar que la disminución de la OC se asocia a una mayor DMO y el análisis de las curvas ROC sugiere que la OC puede ser una herramienta de cribado útil para seleccionar pacientes diabéticos con probable patología ósea. La OC no solo regula la formación ósea, sino que también ejerce otras funciones en otros tejidos, como el páncreas y el tejido adiposo, entre las que se encuentran la regulación de la glucosa y el metabolismo energético. La OC está regulada por varias hormonas, como la insulina, que se une a los osteoblastos y provoca la secreción de OC, que a su vez promueve la proliferación de células β y el aumento de la secreción de insulina, contribuyendo así al control osteometabólico [22]. Nuestro hallazgo de que los niveles de OC se correlacionan significativa y negativamente con la HbA_1c_ en la T1D y con la glucemia en ayunas en la T2D concuerda con esta idea de que la OC funciona como una hormona que regula el metabolismo de la glucosa [23]. La estrecha relación entre la OC y el metabolismo energético, cuya regulación se ve afectada en la DM, indican la necesidad de establecer un punto de corte inferior para la OC para la detección de alteraciones óseas en individuos con DM. De este modo, proponemos un punto de corte inferior de OC para los pacientes diabéticos que podría ayudar a los especialistas a identificar a los pacientes con mayor riesgo de sufrir pérdida de masa ósea.

### Asociación entre los polimorfismos en el gen *VDR* con el metabolismo óseo

Se investigó la asociación entre varios SNP en el gen *VDR* y el riesgo de osteoporosis. Los datos obtenidos muestran que *ApaI* fue el único SNP asociado con la DM. Estos resultados concuerdan con estudios previos en los que se analizaron los polimorfismos del *VDR* en diferentes tipos de diabetes [24], [25], [26]. Los resultados del análisis de las frecuencias genotípicas de *BsmI* y *FokI* obtenidos en nuestro estudio son muy similares a los informados por Ji y col [27]. en una población caucásica y en dos poblaciones españolas [28], en los que no se observaron diferencias en las frecuencias genotípicas de *BsmI* y *FokI* entre el grupo de control y los pacientes con T1D. Sin embargo, otros estudios realizados en otros grupos étnicos muestran una asociación entre los polimorfismos en *VDR* y la DM [29, 30].

Observamos una asociación entre los alelos de los polimorfismos en *BsmI*, *ApaI* y *TaqI* y la DMO lumbar, y de todos los polimorfismos estudiados con los niveles plasmáticos de OC. Estos resultados coinciden con los obtenidos en el meta-análisis realizado por Thakkinstian y col. [31], Jia y col. [32], un estudio en población pediátrica [33] y los publicados por Álvarez-Hernández y col. [10], donde la presencia del genotipo A/A de *BsmI* comparado con la combinación de los otros dos genotipos se asoció a niveles reducidos de DMO lumbar. Dicho estudio concluye que los genotipos G/G de *BsmI*, C/C de *ApaI* y C/C de *TaqI* están asociados a niveles superiores de DMO lumbar y niveles circulantes de OC elevados. Nuestro estudio respalda dichos hallazgos, a excepción de *TaqI*, donde obtuvimos resultados opuestos, esto es, valores inferiores de DMO lumbar y OC en presencia del alelo C de *TaqI*. Estas diferencias en *TaqI* pueden ser debidas a la población seleccionada, ya que existe gran variabilidad en los resultados de los estudios sobre la influencia de los SNP *VDR* sobre la DMO y los niveles de MRO [13]. Esto podría explicarse por el desequilibrio del ligamento, tamaños de muestra insuficientes, o la heterogeneidad de las poblaciones estudiadas. Además, la mayoría de los estudios se han realizado en mujeres premenopáusicas y posmenopáusicas, habiendo pocos datos disponibles en hombres. Una de las fortalezas del presente estudio es que, al incluir a hombres diabéticos en la muestra, completamos los resultados hasta ahora existentes.

A la luz de los resultados obtenidos, los genotipos *BsmI*, *ApaI* y *TaqI* están potencialmente asociados a la DMO en pacientes diabéticos, aunque no parecen ser específicos de enfermedad diabética, ya que la distribución de frecuencias no difiere de la de la población general. Debido al efecto negativo de ciertos alelos sobre la DMO y la OC, el presente estudio indica que los polimorfismos en el gen *VDR* podrían contribuir a la pérdida ósea en pacientes diabéticos. No obstante, es preciso dilucidar en futuros estudios la utilidad clínica de estos hallazgos.

Este estudio presenta algunas **limitaciones**. En primer lugar, no se evaluó la calidad ósea mediante el análisis qCT o *Trabecular Bone Score*. Así mismo, se empleó un tamaño de muestra limitado para el análisis genético. Finalmente, se trata de un estudio trasversal, por lo que no se realizó un seguimiento de los MRO para evaluar la posible utilidad clínica de la OC en el seguimiento de los pacientes.

En **conclusión**, los datos obtenidos en el presente estudio revelan que no existen diferencias en la DMO entre los pacientes con T1D y T2D y que la OC podría ser un marcador candidato para el cribado de la pérdida ósea en pacientes diabéticos. El elevado valor predictivo negativo del punto de corte de la OC indica que ésta podría ser útil a la hora de descartar el riesgo de sufrir pérdida ósea, permitiendo ofrecer un abordaje clínico personalizado para prevenir esta patología. Son necesarios futuros estudios para validar estos nuevos puntos de corte asociados a la calidad ósea.

## Supplementary Material

Supplementary Material

## Material Suplementario

Este artículo incluye material suplementario (https://doi.org/10.1515/almed-2023-0158).
