# Supplementary material for: La osteocalcina se asocia con la densidad mineral ósea y los polimorfismos del gen VDR en la diabetes tipo 1 y 2
Source: Adv Lab Med. 2023 Dec 13;5(1):56–65. [Article in Spanish] doi: 10.1515/almed-2023-0158 (PMC11019893; doi:10.1515/almed-2023-0158)
Supplement: Supplementary file 1 — Supplementary Material [file j_almed-2023-0158_suppl_001.doc]

**Material Suplementario - Tabla 1**Asociación entre los polimorfismos en el gen *VDR*, la DMO y los MRO

| ***VDR/BsmI*** | **Genotipo**  **GG** | **Genotipo**  **AG** | **Genotipo**  **AA** | ***p*** | **Alelo G** | ***p*** | **Alelo A** | ***p*** |
| --- | --- | --- | --- | --- | --- | --- | --- | --- |
| *T-score* cadera, DE | -0,01±1,06 | -0,22±1,09 | 0,27±1,72 | ns | -0,12±1,06 | ns | 0,06±1,32 | ns |
| *T-score* lumbar, DE | 1,36±1,68 | 0,51±1,49 | 0,47±1,70 | ns | 0,83±1,60 | ns | 0,50±1,54 | 0,03 |
| *Z-score* cadera, DE | 0,33±0,45 | 0,72±1,10 | 0,47±1,15 | ns | 0,61±0,97 | ns | 0,64±1,09 | ns |
| OC (µg/L) | 14,41±6,91 | 16,21±8,69 | 12,00±6,68 | ns | 15,45±7,98 | 0,044 | 14,83±8,27 | ns |
| β-CTX (µg /L) | 0,28±0,17 | 0,34±0,26 | 0,19±0,09 | 0,027 | 0,31±0,23 | 0,016 | 0,30±0,23 | ns |
| P1NP (µg /L) | 42,62±24,52 | 48,29±37,00 | 34,58±19,63 | ns | 45,92±32,30 | ns | 43,86±32,88 | ns |
| ***VDR/ApaI*** | **Genotipo**  **CC** | **Genotipo**  **AC** | **Genotipo**  **AA** | ***p*** | **Alelo C** | ***p*** | **Alelo A** | ***p*** |
| *T-score* cadera, DE | 0,55±1,55 | -0,28±1,05 | 0,07±1,24 | ns | -0,18±1,09 | ns | 0,38±1,25 | ns |
| *T-score* lumbar, DE | 0,44±1,71 | 0,62±1,30 | 1,53±1,98 | ns | 0,85±1,53 | ns | 0,56±1,43 | 0,04 |
| *Z-score* cadera, DE | 0,41±1,14 | 0,67±1,07 | 0,59±0,40 | ns | 0,65±0,97 | ns | 0,56±1,08 | ns |
| OC (µg/L) | 13,0±6,04 | 14,05±5,02 | 9,90±3,21 | 0,031 | 13,73±5,29 | 0,011 | 12,95±4,86 | ns |
| β-CTX (µg /L) | 0,25±0,16 | 0,32±0,26 | 0,25±0,16 | ns | 0,29±0,22 | ns | 0,29±0,23 | ns |
| P1NP (µg /L) | 39,00±20,96 | 47,84±36,82 | 37,59±16,77 | ns | 44,63±32,14 | ns | 44,89±32,50 | ns |
| ***VDR/TaqI*** | **Genotipo**  **TT** | **Genotipo**  **TC** | **Genotipo**  **CC** | ***p*** | **Alelo T** | ***p*** | **Alelo C** | ***p*** |
| *T-score* cadera, DE | 0,01±0,98 | -0,32±1,17 | 0,65±1,73 | ns | -0,17±1,09 | ns | -0,07±1,36 | ns |
| *T-score* lumbar, DE | 1,27±1,54 | 0,37±1,56 | 0,82±1,78 | ns | 0,73±1,60 | ns | 0,49±1,61 | 0,046 |
| *Z-score* cadera, DE | 0,43±0,47 | 0,78±1,13 | 0,27±1,10 | ns | 0,67±0,98 | ns | 0,63±1,11 | ns |
| OC (µg/L) | 14,05±5,82 | 12,86±4,34 | 8,97±2,87 | 0,021 | 13,40±5,05 | 0,006 | 11,78±4,32 | ns |
| β-CTX (µg /L) | 0,28±0,17 | 0,32±0,26 | 0,20±0,10 | ns | 0,30±0,22 | ns | 0,28±0,23 | ns |
| P1NP (µg /L) | 43,3±24,2 | 46,6±36,5 | 36,9±22,1 | ns | 45,11±31,61 | ns | 44,06±33,48 | ns |
| ***VDR/FokI*** | **Genotipo**  **TT** | **Genotipo**  **TC** | **Genotipo**  **CC** | ***p*** | **Alelo T** | ***p*** | **Alelo C** | ***p*** |
| *T-score* cadera, DE | -0,34±1,19 | 0,04±1,24 | 0,89±1,99 | ns | 0,10±1,27 | ns | -0,14±1,21 | ns |
| *T-score* lumbar, DE | 0,72±1,77 | 0,51±1,44 | 1,38±1,17 | ns | 0,58±1,42 | ns | 0,62±1,61 | ns |
| *Z-score* cadera, DE | 0,46±0,90 | 0,58±1,19 | 1,77±0,10 | ns | 0,69±1,18 | ns | 0,51±1,00 | ns |
| OC (µg/L) | 15,92±8,95 | 14,32±7,18 | 11,13±0,89 | ns | 13,79±8,94 | ns | 15,18±8,17 | 0,004 |
| β-CTX (µg /L) | 0,32±0,26 | 0,26±0,15 | 0,17±0,08 | ns | 0,24±0,14 | ns | 0,29±0,21 | ns |
| P1NP (µg /L) | 47,94±38,28 | 40,24±22,71 | 35,61±5,95 | ns | 39,49±20,92 | ns | 44,38±32,08 | ns |

Nota: Los datos se presentan como media ± DE

*p* para la diferencia entre los diferentes genotipos y alelos

DE: desviación estándar; ns: no significativa.
